# Perspectives in musculoskeletal injury management by traditional bone setters in Ashanti, Ghana

**DOI:** 10.4102/ajod.v4i1.97

**Published:** 2015-07-16

**Authors:** Anthony K. Edusei, Frances E. Owusu-Ansah, Joslin A. Dogbe, Julia Morgan, Kofi Sarpong

**Affiliations:** 1 Department of Community Health, School of Medical Sciences, Kwame Nkrumah University of Science and Technology, Ghana; 2 Department of Behavioural Sciences, School of Medical Sciences, Kwame Nkrumah University of Science and Technology, Ghana; 3 Department of Child Health, School of Medical Sciences, Kwame Nkrumah University of Science and Technology, Ghana; 4 Honorary Lecturer, School of Public Health (Laureate Online Education), University of Liverpool, United Kingdom; 5 The Samuel Wellington Botwey Foundation (SWEB) Foundation, Ghana

## Abstract

**Background:**

The popularity of the services of traditional bone setters (TBS) in Ghana as an alternative health care requires exploration and documentation of the perspectives of providers and users.

**Objective:**

To explore and document the perspectives of providers and users of the services of TBS in the management of musculoskeletal injuries in the Ashanti region, Ghana.

**Methods:**

From the social constructivist and qualitative approach, in-depth interviews were used to explore the perspectives of eight TBS and 16 users of their services, selected purposively through snowballing. Thematic content analysis (TCA) was employed.

**Results:**

High recovery rate, warm reception, prompt attention, and the relatively lower charges, are reported to motivate the patronage of the services of TBS for the management of fractures in the legs, arms, ribs, joint bones dislocations, waist and spinal cord problems. The TBS combined traditional and orthodox procedures, using plant and animal-based materials, beliefs, spirituality (God-given) and physical therapy in the management of musculoskeletal injuries. No adverse experience was reported by either the providers or users of the traditional management methods.

**Conclusion:**

With plant and animal-based materials, TBS are observed to combine traditional and orthodox procedures to confidently manage musculoskeletal injuries to the satisfaction of their highly motivated patrons. Although over 60% of the TBS attribute the healing power behind their practice to God, the rest do not discount the role of spiritual therapy. Further studies expanded to include the perspectives of non-users of the services of the TBS will authenticate the findings of this study.

## Introduction

Injuries are a public health problem in developing countries (Mock, Forjuoh & Rivara 1999), with an anticipated increased rate and associated disabilities (Murray & Lopez 1996) as a result of increased use of motorised transport. Indeed, the human suffering emanating from injury-induced disability (Mock *et al.* 2003) in developing countries calls for a critical appraisal of the management strategies of musculoskeletal injuries, including those of traditional bone setters (TBS), as a result of the high patronage of such services because of the relatively lower cost, familiarity, and the carried notion of their indispensability or desirability (Ariés Mercel *et al.* 2007; Dada *et al.* 2009; Dada, Yinusa & Giwa 2011; Nwachukwu *et al.* 2011; Nwadiaro *et al.* 2008; Onuminya 2006; OlaOlorun, Oladiran & Adeniran 2001; Thanni 2000), despite reported associated complications such as non-union, malunion, extremity gangrenes, osteomyelitis, and sepsis (Dada *et al.* 2009, 2011; Onuminya 2004).

The major causes of injuries in Ghana as established by Mock *et al.* (1999) are road traffic accidents, burns and falls. Segregating the data into urban and rural areas, Mock *et al.* (1999) have also reported that in both locations transport-related injuries are the leading mechanism of injury, accounting for 16% of all urban injuries and 10% of all injuries in the rural areas, with male predominance and the younger ages (< 15 years) and older ages (> 45 years) mostly affected. Disability resulting from transport-related injuries are, however, higher in older adults (Mock *et al.* 1999).

Transport-related injuries which rank topmost among the injuries requiring the services of TBS are reported to be more severe than the other types of injuries in terms of the associated mortality, length of disability and economic hardships (Mock *et al.* 1999).

Normally, the hospital is the first point of call for primary fracture management, but in Ghana and elsewhere on the African continent many injured persons never seek formal medical care (Dada *et al.* 2011; Nwachukwu *et al.* 2011; Nwadiaro *et al.* 2008; Onuminya 2006; OlaOlorun *et al.* 2001; Mock *et al.* 1999). Ariés Mercel *et al.* (2007) in a study on fracture treatment by bone setters in the central region of Ghana established that over a period of three months, 14 patients diagnosed with fracture left hospital to seek treatment by bone setters. Classified among these traditional healers were persons who use herbs, animal and mineral substances or other methods, and priest-healers who use supernatural powers to provide healthcare.

The World Health Organisation (WHO) defines traditional medicine as:

… the sum total of the knowledge, skills and practices based on the theories, beliefs and experiences indigenous to different cultures, whether explicable or not, used in the maintenance of health, as well as in the prevention, diagnosis, improvement or treatment of physical and mental illnesses. (WHO 2008)

And a traditional healer as:

… a person who is recognised by the community where he or she lives as someone competent to provide health care by using plant, animal and mineral substances and other methods based on social, cultural and religious practices. (WHO 2000)

Before the introduction of orthodox medicine in Africa, the continent's traditional medicine existed and has since been widely practised (Abdullahi 2011; Fokunang *et al.* 2011) and promoted by traditional healers (Ariés Mercel *et al.* 2007; Kennedy 2011).

African traditional medicine which is known to be the major type of health care for many (Abdullahi 2011; Fokunang *et al.* 2011; Kennedy 2011) is described as a holistic treatment achieved through a combination of spiritual beliefs, herbs, animal and mineral products (Abdullahi 2011; Kennedy 2011). Fokunang *et al.* (2011) indicates further that the practice is based on knowledge and beliefs, incorporating spiritual therapies, manual techniques and exercises, applied singularly or in combination. These knowledge and skills are passed on from generation to generation (Kennedy 2011).

Another important feature of African traditional medicine is its ethnocentric, broad and varied nature (Abdullahi 2011). Evidence also abounds to establish the link of spirituality with African traditional medicine. The African peoples’ worldview is basically spiritually oriented, with no clear demarcations between the sacred and the secular. Spirituality permeates all aspects of the African way of life. Kennedy (2011) sums it up by the assertion that spiritual belief such as witchcraft influences African traditional medicine, and, because most of the native people attribute disease causation to witchcraft, attempts at healing by traditional healers would begin with a determination of the cause. In many African cultures, diseases which fail to respond to initial treatment are stigmatised in the society, and believed to be caused by supernatural powers and are therefore reported to the traditional healers for treatment (Abdullahi 2011). This observation gives credence to the fact that traditional African medicine is aligned with spirituality.

As a country with overreliance on traditional medicine and limited healthcare workers as a result of brain drain (Friedman 2004; WHO 2008), yet overburdened with an increase in traumatic injuries because of rapid urbanisation and the growing dependence on motor vehicles (Ariés Mercel *et al.* 2007; Quansah, Afukaar & Salifu 2001), Ghana needs a critical evaluation and documentation of the traditional management of musculoskeletal injuries by TBS who remain a valuable health care resource for many. For this purpose, this research sought to answer two key questions: ‘What are the beliefs, methods, experiences and the principles applied by TBS in the management of musculoskeletal injuries in the Ashanti region of Ghana?’ and ‘What are the perspectives of users of the services of TBS in respect of their beliefs, experiences and the reasons regarding the use of such services?’

Therefore the specific objectives of the study were to ascertain through in-depth interviews the beliefs, methods, experiences and principles applied by the TBS in the Ashanti region of Ghana in the management of musculoskeletal injuries, whilst assessing the beliefs, experiences and reasons for the use of the services of TBS in the management of musculoskeletal injuries in the study area. The findings of the study will contribute significantly to augment the limited publications on traditional medicine in Ghana. Furthermore, the objectives of the WHO Congress on Traditional Medicine held in Beijing, China from 7‒9 November 2008 to share information and experience on research, education, and practice of traditional medicine, and to promote the proper use of traditional medicine will be achieved (WHO 2008).

## Methods

### Study setting

The study area was the Ashanti region, the most populous and most centrally positioned among the 10 regions of Ghana. The total land area of the region is approximately 24 390 square kilometres representing 10.2% of the land area of Ghana (Ghanaweb 2012). As the most populous region, the population forms 19.1% of the total population of Ghana with females constituting 49.8%, as per the 2000 population Census. The majority (51.3%) of the people in the region live in urban settlements.

Ashanti region has won for itself the accolade of ‘centre of culture’ and has 33 traditional councils. In terms of traditional authorities, each council is headed by a paramount chief who is the custodian of the culture. All the paramount chiefs owe allegiance to the Asante king, Otumfuo the Asantehene, who doubles as the head of the Asanteman council as well as the head of the Kumasi Traditional Council (Ghanaweb 2012). The region is endowed with rich natural resources and people with high entrepreneurial acumen who engage in economic activities like subsistence food crop farming, cash crop (cocoa) farming, petty trading, and artisanal work in the construction and mechanical industry. Although almost all the ethnic groups in Ghana are represented in the Ashanti region because of its central position and its rich natural resources, the dominating ethnic group are the Akans, with Twi as the widely spoken language. The high doctor to patient ratio of one to 40 000 people (Ministry of Health [MoH] 2000) contributes to the continued overreliance of people on ‘traditional’ medical care (Ariés Mercel *et al.* 2007).

## Research design

The study adopted the qualitative approach, using an in-depth interview for a better understanding of the perspectives of TBS and users of their services.

### Piloting

Recognising the importance of piloting in any field research (Polit, Beck & Hungler 2001; Teijlingen & Hundley 2001) the interview schedule was piloted on one provider and one user of TBS, and subsequently modified for the main study.

### Sampling

Eight TBS and sixteen users of their services were selected using purposive and ‘snowball’ sampling, that is, asking the whereabouts of another person who provides such services (Heckathorn 2002): following the identification of the first TBS, the others and their patrons were selected through snowballing. As the study was purely qualitative meant to ‘discover’ rather than ‘measure’ perspectives on traditional medical practice, random sampling and sample size were not considered critical (DePaulo 2000), although a theoretical sampling approach (Green & Thorogood 2009) was used to reach the sample sized used. Potential interviewees had to be searched for.

#### Data collection

The in-depth interviews were administered by the researcher, using the interview schedules as a guide. Each interview was preceded by the provision of the research background by reading and interpreting the information sheet, followed by the signing of the consent form. After consent had been given, the interview took place separately for each TBS and two users of his or her services, maintaining some level of privacy to avoid one influencing the other. The proceedings of the interviews were recorded with a digital voice recorder (VR-P2340NEW). The researcher began the interview by making notes on gender, age and non-verbal communications and personal observations about the interviewee. For the users of the services of the TBS, the parameters examined were beliefs, experiences and reasons for using TBS; whilst for the TBS, the parameters covered were beliefs, methods, experiences and principles applied in the management of the musculoskeletal injuries.

#### Data management and analysis

The recorded interviews on the digital voice recorder were kept under lock and key and later transcribed. Using thematic content analysis (Aronson 1994; Green & Thorogood 2009; Marks & Yardley 2004), the transcribed interviews were analysed by first coding them according to the responses to the key questions, categorisation and subsequent grouping under the broad thematic areas for the identification of inherent patterns.

#### Ethical considerations

Ethical approval to establish the non-invasive nature of the study was sought from the Committee Human Research and Publications Ethics (CHRPE) of the School of Medical Sciences (SMS) and Komfo Anokye Teaching Hospital (KATH), Kwame Nkrumah University of Science and Technology (KNUST), Ghana, and the University of Liverpool Committee on Research Ethics. Following the approval of the CHRPE in Ghana on 16 May 2012 approval from the University of Liverpool was also obtained.

Following the initial contacts for recruitment in the study and the subsequent appointment with them for interviews, the interviewees were informed about the research and given consent forms to sign to confirm their readiness to be interviewed. They were assured of the confidentiality of the information being sought.

## Trustworthiness

### Interview tools and handling of data

In-depth interviews were conducted separately for the eight TBS and 16 users of their services, using schedules prepared on the basis of the objectives of the research. The interviews were conducted by the researcher without any interpreter. With the permission of the interviewee, the interviews were recorded using a digital voice recorder (VR-P2340NEW).

### Validity and reliability

Reliability and validity are important characteristics of qualitative research (Green & Thorogood 2009), with reliability essential in determining the validity of the data (Shuttleworth 2008). The reliability and validity of the information generated was ensured as follows: interviews were conducted and recorded using a digital voice recorder and transcribed by the researcher, writing notes where possible, and comparing the data with transcripts to validate information captured.

## Results

### Perspectives of the traditional bone setters

#### The practice of traditional bone setters

This broad theme examines issues related to initiation and experience in the practice, diagnostic and therapeutic procedures, the healing experience, principles and the healing powers behind the practice. All the TBS reported that their practice is family-based, and a gift from God handed over from one generation to another within the family:

‘The business [*traditional bone setting*] was not learnt through apprenticeship. It is a gift from God for the family. God gave it to my grandfathers. My father came to continue, and now it is our turn to continue’. (P6)

The common musculoskeletal injuries the TBS manage are fractures in the legs, hands, ribs, collar bones, spinal cord and waist bones, joint dislocations, and neck bone injuries. Only two TBS indicated their ability to treat head bone injuries, although with caution, provided the victim has received the attention of a medical officer, and has been certified not to be in any life-threatening situation. The part-time bone setters, apprehensive of the legal implications of complications resulting from their management, however, restrict their management to only injuries in the legs and arms: ‘Exactly! The hand, the legs, the spinal cord, ribs; as long as it is a case of bone fracture, even if it is the skull, we manage it’ (P3):

‘I treat mostly the fractures in the hands and legs. As I have told you I am not doing the work for full-time, so I don’t try any complicated case’. (P8)

It is noteworthy that all the TBS interviewed demonstrated confidence in their healing capabilities for any injury case for which they were satisfied through their investigations that the cause is not attributable to supernatural powers or witchcraft. All of the TBS were candid enough to indicate that they would refer road traffic accident victims first to the hospital for emergency treatment before they will attempt management with traditional methods to avoid complications. They all ascribe the healing power behind their treatment to God, although one of them did not ignore the role of ‘spiritual powers’: ‘I am very confident about what I am doing, because I am not afraid of any type of injury presented to me for management’ (P6); ‘For us our backbone is God. We have nothing like some spiritual powers which are giving us directions as to what to do’ (P3); ‘As for that I will say we have no supernatural powers backing us. We are healing people through God's healing powers’ (P4):

‘Spiritual backing is required in the practice because not all injuries occur by accident, some are caused by evil forces, and you need the spiritual backing to treat, otherwise the demons can disturb you as a healer’. (P5)

#### Diagnostic procedures and its rationale

The study revealed that more than half of the TBS use conventional medical approaches such as diagnosis before treatment begins, and use conventional bandage and latex gloves, and pain-killers. The diagnostic procedures include enquiries into the cause of the injury, touching and feeling with the fingers to ascertain the nature and severity of the fracture, and the use of X-ray photographs to ascertain the nature and anatomical site of the fracture. Further enquiries into these practices established that it is in response to recommendations of health workers who patronise the services of the TBS, or skills acquired through long years of experience.

The rationale behind the initial investigations is to ascertain the nature and extent of injuries, to ensure that they do not entertain cases of road traffic accidents that may suffer internal bleeding which is beyond their capabilities, and to avoid providing treatment for thieves who have suffered gunshot wounds. ‘For an accident victim, if the doctor has not proven beyond doubt that there has not been any internal bleeding, I will not attempt to handle such a person’ (P1).

#### Therapeutic materials used:

The therapeutic materials used by the TBS appeared universal among all of them, except four who gave indications that besides water and Shea butter they use plant or animal-based preparations. Three respondents were adding a black powdery substance to the Shea butter before applying the ointment to the injured parts for massaging. One said it was tortoise shell which is burnt and ground into powder. Two others confirmed using plant parts which are also burnt and supplied from the northern part of Ghana. Enquiry on the reason for the use of the Shea butter or lard and water revealed that it was to facilitate the physical therapy (massaging) aspect of the management of the injury. With respect to the animal or plant-based preparations, one TBS used the analogy of welding two metal pieces with electrodes to indicate it is used as the active binding substance:

‘We use tree parts and even animal parts (tortoise). You can burn the case of the tortoise to char and grind it into powder and mix it with animal lard (from snake or cattle), or Shea butter and apply to the injured parts’. (P5)

‘As for the medicine, I am not in a position to tell you anything about it. Moreover, it is not prepared here. It is brought from the northern part of Ghana for us to use. It is a black powdery substance prepared from a burnt tree root’. (P1)

The other materials used by all the TBS are wooden splints and little mats prepared from palm fronds. To avoid shifting and subsequently malunion or non-union of the broken bones the TBS use these materials to support and stabilise the fractured bones, particularly the single bones of the upper arm and the thigh after setting them.

#### Therapeutic procedures

Generally, therapy begins with the rubbing of the affected part with warm water, followed by the application of the Shea butter (with or without herbal or animal preparation) and massaging, then bandaging (with a wooden splint or mat support if necessary). The nature, length of stay of the bandaging depends on the nature of the injury and the affected bone; the single bones like the thigh and upper arm bones which are not frequently disturbed, stay longer. Apart from one respondent who would offer treatment four times, morning and evening for four days for women, and only two days for men, all the other TBS would provide continuous and daily treatment from the start until the injured is totally healed. The explanation provided by the TBS for the difference in the treatment period for women and men was that increased activity and the softer tissues on the part of the woman may disturb the bandaged parts, thus requiring more frequent attention:

‘If you are a female I give you treatment for four times; morning and evening for four days. If it is a male who presents any degree of fracture, I will provide treatment for only two days; morning and evening of both days, and that is all’. (P7)

### Spirituality and traditional bone setting

Although all the TBS attributed their therapeutic powers to God, and possibly their skills in massaging regularly to set the fractured bones, they did not rule out spirituality in the cause and treatment of injuries. One TBS indicated that in their family practice they are taken through some spiritual initiation involving the recitation of special prayers which enable them to identify injuries caused by evil forces. He confirmed this assertion with this quote:

‘Yes! Because not all injuries occur by accident; some are caused by the devil, and you need the spiritual backing to treat it otherwise the demons can disturb you the healer’. (P5)

The perception among the TBS is that if the injury is caused by an evil force, healing becomes difficult or almost impossible. To them this is where they need spiritual powers to annul the effect of the evil forces to facilitate the healing process. The TBS were not oblivious of the fact that the general perception carried by most people who are hesitant in accessing their therapeutic services is that they depend on supernatural powers in providing healing. It is in the attempt to disabuse people's minds of this perception that they provide the care in the open.

#### Strengths of the traditional bone setters

Whereas two of the TBS were emphatic in stating that their strength is of divine origin, the strength of the rest apparently lies in their total dedication to work, patience and respect for their patrons, commitment to work, minimal charges, and the high recovery rates which are widely perceived and propagated by their patrons. ‘I will say that I derive my strength from God Almighty. It is in him we derive our being and do everything’ (P8).

#### Weaknesses of the traditional bone setters

In their own estimation, the TBS do not have any weakness in the performance of their duties as reflected in these expressions: ‘I don’t think I have any weakness, safe sickness which will disable me’ (P4).

In addition, participant 7 stated:

I don’t really have any weakness, but if I get any help from any source it will be welcome to enable me provide accommodation for those who come for treatment.

#### Challenges of the traditional bone setters

The major challenges confronting the TBS in the discharge of their duties were the reported inadequate space to operate from, and to provide accommodation for their clients coming from far. Of major concern to them is the unavoidable arrangement to move the injured to and from the healing centre to receive medication, which retards the healing process as the set broken bones may be frequently disturbed.

In the course of providing services, parts of the bandaged limbs become swollen, and this often attracts the condemnation of health workers and some family members, friends or acquaintances of the injured. Another reported challenge is with respect to flesh getting stuck on the surfaces of the fractured bones, thus preventing a perfect contact when the broken bones are joined up, and subsequently making the healing almost impossible. In their view the single bones in the thigh and the upper arm take longer to heal, because there is often lack of proper support for the broken bones in these parts of the body. They also opined that fractures that are caused by evil forces are difficult to heal, and requires a spiritually strong person to detect and address these cases (Table 1).

**TABLE 1:** Background information of users of the services of traditional bone setters.

Age (years)	Gender	Education	Occupation	Injury
54	Female	Basic education	Trading	Neck, ribs, and inner trunk pains as a result of road traffic accident
16	Male	Junior Secondary	Pupil	Twisted right knee injury occurred in a football match
36	Female	Junior Secondary	Food vendor	Fractured lower right leg, and ankle dislocation
26	Female	Polytechnic	Student	Compound fracture in left lower arm
70	Female	No formal education	Food vendor	Dislocated wrist due to fall
55	Female	Basic education	Trader	Dislocated left shoulder from road traffic accident
25	Male	Basic education	None	Twisted hands, legs and waist as a result of road traffic accident
44	Male	Senior Secondary	Civil servant	Persistent severe knee pain, restricting movement
54	Female	No formal education	Trader	Severe knee injury after fall
45	Male	Basic education	Trader	Compound fracture in both legs after being run down by a vehicle
32	Female	University	State registered nurse	Compound fracture in the right arm.
48	Male	No formal education	Farming	Neck injury after a wooden plank has fallen on user.
36	Female	University	Banker	Twisted ankle from a high-heeled shoe
46	Male	Polytechnic	Marketing	Severe waist pain from road traffic accident
43	Female	Senior secondary	Trader	Twisted right leg because of fall
38	Male	Basic education	Carpenter	Broken left lower limbs

### Users’ Perspectives

#### Perceptions about the services of traditional bone setters

The results of the study indicate that positive testimonies regarding the outcome of services provided by TBS, experiences of friends, family members and close acquaintances, dislike for plaster of Paris (POP), perceived longer periods of recovery from the services provided at the orthodox health facilities, motivate people to seek the services of TBS. Most participants patronised the services of the TBS, fully confident that they would receive total healing in much shorter time compared to orthodox medical treatment. Indeed, almost all study participants, except three, had first sought the care of the orthodox health care providers, but ended up with the TBS as a result of the perceived slow healing process; ‘You know the hospital is where we’ll go first, but they could not treat it, and that is why I have brought it here’ (P5.1):

Apart from what I have experienced and witnessed myself, many have also sought the services of the TBS and are full of admiration and praises because they received their healing. (P6.3)

One significant revelation from the study is that all the participants reported experiencing significant improvement in their situation within one month of accessing the services of the TBS. ‘Because my arm was dislocated, I suffered severe pains, but since the first day she managed to put it back into the socket, the pains went down drastically’ (P3.2).

##### The healing procedures and methods of the traditional bone setters

With respect to the procedures and methods used, the participants affirmed what was said by the TBS. They begin with the initial diagnosis through a series of questions, whilst feeling with their fingertips, with some using X-ray photographs to ascertain the type and cause of injury. The purpose, as reported by the users, is for the TBS to evaluate the case bearing their capabilities and skills in mind, and once they were certain they could treat it, they would begin to offer services. The TBS were reported to use water (warm for most of them), Shea butter (a few adding a black powdered substance), and wooden splints or small mats used as support before bandaging. Apart from one lady bone setter, all the TBS would provide daily or regular consultations and service until the injured is totally healed:

‘They use Shea Butter and water, and then bandage. These are just the materials they use to massage, and by God's grace they are able to provide healing to the people’. (P1.1)

##### Users’ perspective of strengths, weaknesses and challenges

In the view of the users of the services of the TBS, their strength lies in low charges, warm reception, patience and respect in dealing with their clients, the acceptance of the practice within the socio-cultural context of the people, and their ability to achieve a positive outcome using simple technology. ‘Oh! They are very good, particularly to come to realise that they do not use any sophisticated technology, yet are able to achieve marvellous results’ (P4.2).

None of the users of the services of the TBS reported anything negative about the services. However, four of them expressed concern about the hygiene situation in the environment in which they operate. In their view, some appreciable level of improvement would be advisable:

Apart from the concerns about the one towel and bucket used for many patients, and the bad practices in treating the sores, and the unclean environment most of them operate from, I don’t think there is any negative thing about the practices of the traditional bone setters. (P3.2)

### Discussion

#### Implications of findings

The study appears to highlight traditional bone setting as an indigenous and indispensable practice (Thanni 2000). The practice and training appears to be preserved by the family (Onuminya 2004; Kennedy 2011) and passed on from generation to generation (Dada *et al.* 2011). The providers and users of the services of the TBS seemed satisfied and confident with the outcome of this traditional injury management method, and were ready to share their experiences with others. A major inference from the study findings is that traditional injury management which is reported to be widely practised (Abdullahi 2011; Fokunang *et al.* 2011) remains valuable and acceptable as an alternative health care.

#### Perspective of providers and users of traditional management of musculoskeletal injuries

The findings from the TBS and users interviewed corroborate the work of Mock *et al.* (1999) that falls and transport-related injuries are the major causes of injuries and disabilities.

The results of the study indicate that the providers and users of the services of the TBS have positive perceptions about the services as a result of the satisfaction they all derive from this alternative healthcare. Several reasons can be assigned to this perception, but perhaps the most important are: the high recovery rate, relatively lower costs, familiarity and cultural acceptability, dislike for the POP, the positive experiences regarding the outcome of the services of the TBS shared by friends, acquaintances and family members, warm reception, respect for the clients and the frequent follow-up, which are also reported in other studies (Ariés Mercel *et al.* 2007; Dada *et al.* 2009, 2011; Nwachukwu *et al.* 2011; Nwadiaro *et al.* 2008; Onuminya 2006; OlaOlorun *et al.* 2001; Thanni 2000). The high recovery rate reportedly achieved by the bone setters may be because of the fact that they would always establish the causes and assess their capability in respect of that particular case before attempting to provide healthcare.

Over 80% (13 out of 16 users) had actually sought orthodox healthcare before consulting the TBS because of slow and discouraging treatment outcome, disproving earlier held notions that in Ghana and elsewhere on the African continent many injured persons never seek the services of the orthodox health care facilities (Dada *et al.* 2011; Nwachukwu *et al.* 2011; Nwadiaro *et al.* 2008; Onuminya 2006; OlaOlorun *et al.* 2001; Mock *et al.* 1999). Indeed, the results of the study by Ariés Mercel *et al.* (2007) in central Ghana which established that over a period of three months 14 patients with various forms of fractures who were receiving orthodox care in the hospitals left for treatment by TBS, is repeated in this study. This seems to affirm the fact that for most people, the TBS is the ‘last stop’ for injury management in Ghana and other African countries.

The TBS revealed that they are in general supportive of the practice where the road-traffic injury cases would first report to and receive some attention at the hospital before coming to them. To them this would reduce the chances of any complication of their management, thereby improving their outcome. Generally, this practice of the TBS, which appears to have developed after several years of experience, is commendable and is indicative of their preparedness to compliment the orthodox healthcare in respect of injury management.

The users of the services of the TBS do not consider the methods and processes of diagnosis and healing invasive, uncomfortable, and cumbersome. They in fact expressed high confidence in the safety and efficacy of the treatment: herein lies the strength of the TBS. Indeed, the study found that the diagnostic and therapeutic procedures employed by the TBS are not at variance with orthodox medical procedures. The physical therapy aspect of the treatment by the TBS may be contributing significantly to the healing process, and that may explain why, although the procedures applied by them appear simple, people are healed. Although the practice of traditional bone setting is reported to be shrouded in mystery and spirituality (Abdullahi 2011; Dada *et al.* 2011; Kennedy 2011) in certain places in Nigeria and elsewhere in Africa – a point also asserted by two of the TBS – the healing power is generally attributed to God. This suggests that in a highly religious country like Ghana, traditional management can be improved and promoted as an alternative health care.

It is significant to note that none of the users of the services of the TBS expressed any negative feelings about the services received in response to a related question, except some concerns in relation to hygiene in their premises and practice raised by four out of the 16 participants. The major challenge which needs consideration by policy makers concerns the matter of accommodation which was raised by both the TBS and the users of their services.

#### Public health relevance of the research

The findings of this study can help the government of Ghana to develop a good public policy which can incorporate and strengthen the services of TBS (Naidoo & Wills 2009). The high recovery rate and patronage reported in this study should motivate further investigations into traditional medicine in Ghana to generate more information to facilitate the total integration into the national healthcare system as being advocated by the WHO (2008).

The results of the study provide useful information regarding the services provided by TBS in the management of musculoskeletal injuries by complementing the knowledge in this practice, thereby providing the basis to educate and encourage members of the public who remain sceptical about the safety and value of their services. At the same time, the information obtained should inform the health professionals who have little or no regard and recognition for traditional medicine, and for that matter traditional bone setting as an alternative healthcare, to begin to acknowledge and appreciate its practice in the overall health care system of Ghana.

#### Limitations of the study

This research targeted a limited number of the TBS and the people using their services at the time of the study. For this reason, it could not capture users of such services who might have discontinued as a result of complications or disappointments. It is therefore recommended that further studies should take the form of a community-based survey that will elicit the views of the community regarding this indigenous practice from a broader perspective. In addition, the inclusion of a control group (persons who used only the orthodox medical services) can facilitate a more objective comparison of outcome, patient satisfaction, and possible complications in future studies.

#### Recommendations

Based on the perceived good outcome of the services rendered by the traditional bone setters, the ministry of health of Ghana should support a more comprehensive research into traditional medicine for the purpose of its integration into the national healthcare delivery system in accordance with the recommendation by the WHO (2008).

### Conclusions

This qualitative research has revealed that TBS combine traditional and orthodox procedures, using plant and animal based materials to manage musculoskeletal injuries. Expressing confidence and satisfaction, the TBS and users of their services perceive the traditional method of managing musculoskeletal injuries as good because, in their view, the method is safe and effective. As opined by the users of the services of the TBS, patronage is motivated by the low costs, high recovery rate, respect and tender care. With encouragement from friends, relatives, acquaintances and neighbours or community members who have experienced the services of TBS, the injured access their services confident of being healed.

In-depth studies that will involve non-users to capture the broader perspectives of community members are recommended to enhance the value of the information generated. Furthermore, results of such studies can complement the limited information on TBS.
